# Editorial: Advances in Biological Understanding of Tumor Radiation Resistance

**DOI:** 10.3389/fonc.2020.00754

**Published:** 2020-05-12

**Authors:** Ira Ida Skvortsova, Paul N. Span

**Affiliations:** ^1^Laboratory for Experimental and Translational Research on Radiation Oncology (EXTRO-Lab), Department of Therapeutic Radiology and Oncology, Innsbruck Medical University, Innsbruck, Austria; ^2^Tyrolean Cancer Research Institute, Innsbruck, Austria; ^3^Radiotherapy and OncoImmunology Laboratory, Department of Radiation Oncology, Radboud University Medical Center, Nijmegen, Netherlands

**Keywords:** radiotherapy, treatment resistance, DNA damage repair, hypoxia, cancer stem cells

Radiation therapy (RT) is a frequently-applied powerful treatment approach generally leading to enhanced local tumor control. However, clinical outcome may be compromised by ineffective eradication of cancer cells that exhibit intrinsic or acquired radiation resistance. Although with increased doses of irradiation these radioresistant carcinoma cells can successfully be killed, it is usually impossible to reach these doses without pronounced damage to healthy tissue. To enhance the efficacy of RT, it is important to elucidate the molecular mechanisms regulating radiation resistance of tumor cells. The development of novel chemo- or targeting therapeutics targeting these mechanisms may thereby improve RT efficacy.

A number of mechanisms are known to be involved in cancer cell insensitivity to irradiation. Among them are mutations in genes related to DNA damage and repair, activation of intracellular pro-survival signaling pathways, affected cell cycle regulation, compromised cell death machinery, etc. Furthermore, microenvironmental factors are also very much involved in tumor cell radioresistance. For example, hypoxia, tumor-associated fibroblasts, immune cells, etc. could diminish tumor responses to ionizing radiation. Additionally, cancer stem cells (CSC) encapsulate in a single concept many of the above-mentioned explanations of tumor insensitivity to cytotoxic radiotherapy. Therefore, the role of CSCs in tumor formation, development and response to anti-tumor therapies is at present under intense investigation.

In this Special Issue, several reviews and original research papers address these different mechanisms of radioresistance.

For example, targeting of the repair of RT-induced Double Stranded Breaks in DNA may enhance RT efficacy (Biau, Verrelle et al.), for example via the DNA repair inhibitor Dbait (Biau, Berthault et al.).

Considering the association of intracellular signaling pathways and immunity with radioresistance, (Liu and Sidi) discuss how specific Innate Immune Kinase IRAK1 inhibitors might attenuate tumor radioresistance, while enhancing innate anti-tumor immune responses. Furthermore, Rødland et al. found that the dual-specific CDK1/2 and AURA/B kinase inhibitor JNJ-7706621 inhibits resistance to radioimmunotherapy in Diffuse Large B Cell Lymphoma. A role for the gene Schlafen11 (SLFN11) in determining cancer cell sensitivity to DNA-damaging chemotherapeutic agents and patient outcomes for several cancers has been described. Kaur et al. shows that CD47 is involved in this SLFN11-associated radioresistance.

The tissue microenvironment (TME) influences radiosensitivity. In the multicentric validation study of van der Heijden et al., (acute and chronic) hypoxia, stem cell-ness, tumor growth, epithelial-to-mesenchymal transition (EMT) and DNA repair were found to be related to locoregional control in chemoradiotherapy treated HNSCC patients. Radiosensitivity is also modified by mechanical cues from the TME as reviewed by Deville and Cordes, and cells communicate radioresistance via exosomes as described in Ni et al.. Reciprocally, RT induces extensive changes in the TME that subsequently contribute to radioresistance, as found in glioblastoma (Seo et al.). This latter effect seems mediated via glioblastoma stem cells. This role of CSCs in radioresistance is discussed by Arnold et al.. Herein, the authors indicate that CSC targeting therapy is relevant for the efficacy of RT, but that correct identification of CSCs and reliable distinction from healthy cells is necessary. Furthermore, Terraneo et al. highlight mechanisms of CSC therapy resistance such as EMT and stemness, and describe novel therapeutic strategies for ovarian CSC. Indeed, Neuropilin-2 (NRP2) is associated with radioresistance in bladder cancer, and in Schulz et al. this is reportedly mediated via effects on EMT.

Lindell Jonsson et al. used liquid chromatography-mass spectrometry (LC-MS) to compare the metabolism between 2 HNSCC cell lines with differing radiosensitivity before and after irradiation, and found important differences that may account for their radiosensivity. Notably, Dadgar and Rajaram review different approaches to assess cellular metabolism, such as two-photon microscopy, diffuse reflectance, and Raman spectroscopy, which yield functional and molecular differences between radiation-resistant and sensitive tumors in response to radiation.

Other reviews consider alternative modes of irradiation as a way of alleviating radioresistance. For example, differences in photon vs. particle irradiation exist, as reviewed by Sato et al.. Furthermore, Ultra-High Dose Rate irradiation (FLASH) appears to have differential effects on normal tissues vs. tumors, making -to a certain extent- higher, more effective doses feasible (Wilson et al.). Moreau et al. finds that FBL-03G, a flavonoid cannabis derivative, radiosensitizes metastatic pancreatic cancer. On the other hand, Bettoni et al. report how in rectal cancer, neoadjuvant chemoradiation can increase intratumoral genetic heterogeneity, thereby leading to an increased risk in more aggressive residual tumors.

Overall, this Special Issue has addressed a multitude of potential mechanisms associated with radioresistance, and report on new targets for sensitizing treatment options. Knowledge on how these different mechanisms are induced and interact, how these occur in different cancers, and how these may be countered, will aid in assessing, preventing and/or targeting radioresistance. As radiotherapy is still one of the most effective and widely applied cancer treatment options, countering radioresistance may have far reaching effects on clinical outcome of many cancer patients.

## Author Contributions

PS wrote the first draft. IS contributed and finalized the manuscript.

## Conflict of Interest

The authors declare that the research was conducted in the absence of any commercial or financial relationships that could be construed as a potential conflict of interest.

